# Association Between Testicular Microlithiasis and Histological Subtype in Testicular Cancer

**DOI:** 10.7759/cureus.29946

**Published:** 2022-10-05

**Authors:** Nezahualcoyotl Gonzaga-Carlos, Francisco Virgen-Gutierrez, Juan Carlos Angulo-Lozano, Maria Fernanda Virgen-Rivera, Miguel Maldonado-Avila, Jorge Jaspersen Gastelum

**Affiliations:** 1 Department of Urology, Hospital General de Mexico, Mexico City, MEX

**Keywords:** male cancer, urologic oncology, ultrasonography, microlithiasis, testicular cancer

## Abstract

Background

There is a clear association between testicular cancer and microlithiasis when there are predisposing risk factors such as the history of germ cell tumors in first-degree relatives, testicular atrophy, Klinefelter’s syndrome, and cryptorchidism. This study aimed to establish an association between microlithiasis and the histological subtype of testicular cancer by analyzing data on Hispanic population.

Methods

A retrospective cohort, longitudinal, comparative, and analytic study was conducted on patients with a confirmed diagnosis of primary testicular cancer. The testicular ultrasounds were checked before any surgical treatment to find microlithiasis. We performed a binary logistic regression to establish an association between microlithiasis and the type of testicular cancer.

Results

A total of 130 clinical files were analyzed. Binary logistic regression showed no association between testicular microlithiasis and the subtype of testicular cancer (p = 0.438, 95% CI: 0.80-1.64). The result of the Pearson chi-square test showed no association (p = 0.184). We also analyzed the association between age and microlithiasis using the one-way ANOVA test (p = 0.82) and the association between age and the dichotomic subtype of testicular cancer (seminomatous and non-seminomatous) using the ANOVA one-way test, which showed no significant association in age and testicular cancer subtype (p = 0.178).

Conclusions

There was no association between testicular microlithiasis and the histological subtype of testicular cancer in our study. As mentioned before, we recommend conducting a more extensive study to provide further scientific evidence to establish a reliable association between microlithiasis and the subtype of testicular cancer since there is a discrepancy in the results of our study with the information previously reported. We encourage the study of characterization of risk factors among ethnic groups as this field has not been explored yet.

## Introduction

Testicular microlithiasis (TM) is a condition characterized by microcalcifications within the seminiferous tubule of the testicle [1]. In most cases, these are incidental ultrasound findings. The prevalence of TM ranges from 0.6% to 9.0%, while the prevalence of symptomatic TM is 4.3%-18.2% [2]. The European Society of Urogenital Radiology defines TM based on the number of observed microliths by fields of view: classic (≥ 5 per field of view), limited (<5 per field of view), and diffuse [3]. The first case of TM was reported in a four-year-old boy in 1970 as an incidental finding on a pelvic X-ray. In 1982, the association between microlithiasis and testicular cancer was reported for the first time [4]. The incidence of microlithiasis was higher in patients with testicular tumors compared with those who did not have tumors (74% vs. 16%, p < 0.05) [5]. TM has a peak incidence around two years of age [2-5].

It has been proposed that the cause of microlithiasis is the absence of phagocytosis in the Sertoli cells of the seminiferous tubules, which produces an accumulation of epithelial cell debris, causing an inflammatory and immune response. The role of this immune response and the relation it has with testicular tumors are not yet known. Testicular self-examination is recommended for patients with any risk factor of developing testicular cancer (family background, intratubular germ cell neoplasia, testicular atrophy, Klinefelter’s syndrome, hypospadias, and cryptorchidism) and the presence of microlithiasis.

Some authors suggest an immediate testicular biopsy in case of presenting microlithiasis and identifiable risk factors such as family history, intratubular germ cell neoplasia, testicular atrophy, Klinefelter’s syndrome, hypospadias, and cryptorchidism [6]. TM is relevant in the presence of a testicular tumor since this finding is usually encountered inside or in the periphery of the tumor [7]. In the pediatric population, microlithiasis in a radiologic result does not seem to be associated with testicular malignancy; in the case of teenagers with microlithiasis and risk factors, a monthly self-examination is recommended [8-10].

Several studies have analyzed the relation between microlithiasis and testicular cancer. The most relevant information was provided in a meta-analysis that included 35,578 individuals [11]. They found that patients with microlithiasis are at a 12-fold higher risk of developing testicular cancer than individuals without microlithiasis (RR = 12.70; 95% CI = 8.18-19.71; p < 0.001) [9,10].

An article studied the relation between microlithiasis and the subtype of testicular cancer, and whether the microliths are associated with the stage of the tumor at the moment of diagnosis [12]. The author suggests that microlithiasis might be positively associated with seminoma (p = 0.03) and negatively associated with embryonal cell carcinoma (p = 0.007). Meanwhile, a relation between the stage of the tumor and the presence of microliths was not found [11-12]. The present study aimed to establish the association between microlithiasis and the histological subtypes of testicular cancer.

## Materials and methods

A retrospective cohort, longitudinal, comparative, and analytic study was conducted in the Mexican General Hospital “Dr. Eduardo Liceaga.” The data were gathered from the clinical files of the patients who had a confirmed histopathologic diagnosis of primary testicular cancer and were seen at urology outpatient care of the hospital between 2015 and 2021. The information was captured in a database.

The pathologic reports were used to determine the different subtypes of testicular cancer. Moreover, the information on each patient was captured in a chart that contained the patient's personal and demographic data, their complete medical history, surgery, lab results, pathology results, images from radiological studies, and physical examination.

Before any surgical treatment, the testicular ultrasounds were examined to look for microlithiasis in the system Carestream Vue Motion version 2020 of the Mexican General Hospital “Dr. Eduardo Liceaga.” Microlithiasis was defined by at least five microliths (from 1-3 mm) characterized as hyperechogenic foci observed inside the testicular parenchyma per field of view.

The statistical analysis was done using the Statistical Package for the Social Sciences (SPSS) version 20.0 for Windows (IBM Corp., Armonk, NY, USA). Exploratory univariate analysis and an analysis of normality for quantitative variables were performed, and Student’s t-test and a one-way ANOVA were used for the same variables.

The variables were categorized as follows:

1. Present microlithiasis

2. Absent microlithiasis

·3 Subtype of testicular cancer:

· Seminomatous

· Non-seminomatous:

o Embryonic carcinoma

o Mixed germ cell tumor

o Choriocarcinoma

To establish the association between microlithiasis and the type of testicular cancer, we performed a binary logistic regression and used the Pearson chi-square test. For the qualitative variables, simple frequencies and percentage distribution were used, and the measures of central tendency (average and standard deviation) were obtained. The alpha error was set at 0.05.

## Results

A total of 165 clinical files with a history of attended testicular cancer were identified in our institution; 35 were excluded because their radiological studies were incomplete, or the histological variant was not precise. The patients included were those with a confirmed diagnosis of testicular cancer who had an ultrasound before being diagnosed with testicular cancer.

A total of 130 clinical records were analyzed; 58 tumors were located in the right testicle and 72 in the left testicle. The average age was 29 ± 9.96 years, and the most frequent histological subtype was germinal mixed cell tumor. Overall, 49 (37.6%) of the patients with testicular cancer had microlithiasis shown by ultrasonography, 52 (40%) of the cases of testicular cancer were seminomatous, and 78 (60%) were non-seminomatous (embryonal carcinoma, mixed germ cell tumor, and choriocarcinoma). Serum tumor markers for testicular cancer of the patients were reported by the mean and standard deviation (Table 1).

**Table 1:** Demographic variables of the patients included in the study. Note: X is the numeric value corresponding to the lab value, and DE is the standard deviation LDH, lactate dehydrogenase; hCG-β, human chorionic gonadotropin subunit beta

Variable(N=130)	
Age (years), X ± DE	29 ± 9.96
Side right/left	58/72
Present microlithiasis, n (%)	49 (37.6)
Absent microlithiasis, n (%)	81 (62.4)
Seminomas n (%)	52 (40)
Embryonal carcinoma, n (%)	8 (6.2)
Mixed germ cell tumor, n (%)	66 (50.8)
Choriocarcinoma (%)	4 (3.0)
LDH (u/L), X ± DE	500.31 ± 773
Alpha-fetoprotein (ng/mL), X ± DE	363.63 ± 1706
hCG β (mUI/mL), X ± DE	216.26 ± 716

**Figure 1:**
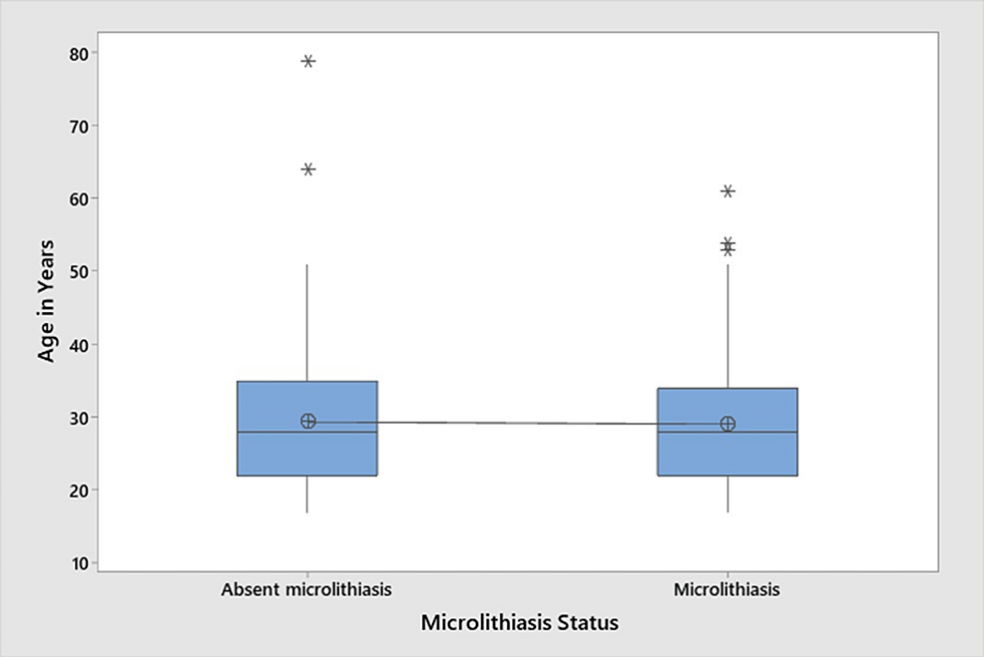
Boxplot of one-way ANOVA test comparing the association between microlithiasis and age (p = 0.820) (n = 130)

We analyzed the association between age and microlithiasis using the one-way ANOVA test, with no statistically significant association between these two variables (p = 0.82), as shown in Figure 1.

**Figure 2:**
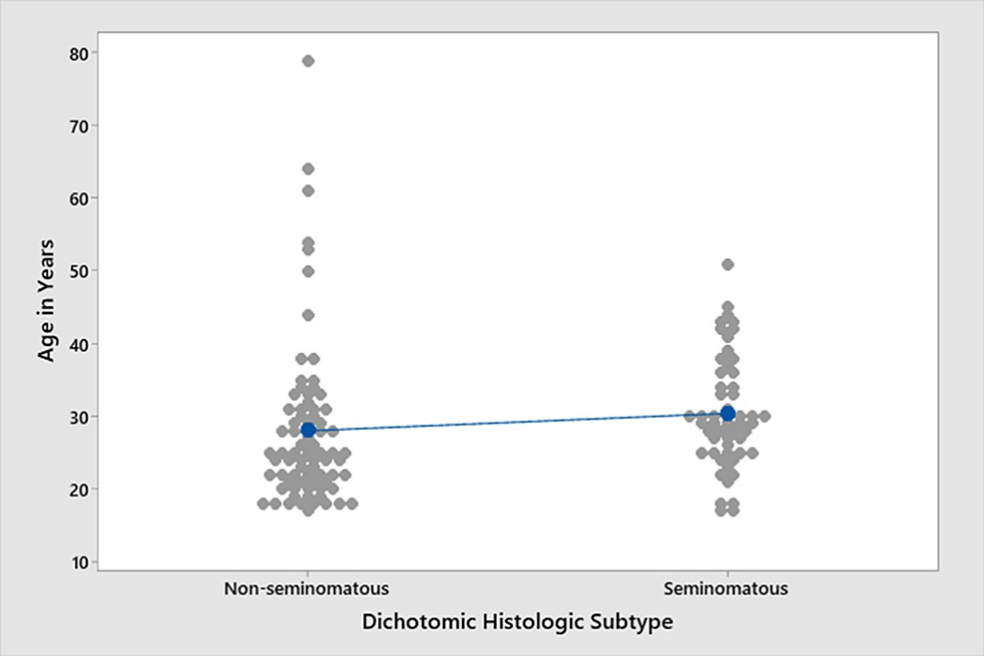
Individual value plot comparing the association between age and non-seminomatous and seminomatous testicular cancer subtype, with non-seminomatous subtype presenting with more variety although not statistically significant (p = 0.178).

We tested the association between age and the dichotomic subtype of testicular cancer (seminomatous and non-seminomatous) using the one-way ANOVA test and found no significant association between age and testicular cancer subtype (p = 0.178), as shown in Figure 2.

Binary logistic regression showed no association between TM and the subtype of testicular cancer (p = 0.438; 95% CI: 0.80-1.64). The result of the Pearson chi-square test showed no association (p = 0.184) (Table 2).

**Table 2:** Association between testicular microlithiasis and histological subtype of testicular cancer. Pearson chi-square *p = *0.184

Variable	Seminomas	Non-seminomas	Mixed germinal	Non-germinal	Total
Present microlithiasis	29	6	45	01	49
Absent microlithiasis	23	2	21	3	81
Total	52	8	66	4	130

## Discussion

The findings of this study demonstrate that there is no relation between the subclass of testicular cancer and the presence of microlithiasis. The results should be interpreted with caution as this is a retrospective study and the study population is limited.

TM incidence varies from 2% to 9% of the populations evaluated by ultrasound [4-6]. There has been controversy in the treatment and follow-up of patients with this condition. Some authors conclude that there is an overestimation of this finding in the asymptomatic population, concluding that 98.4% of men diagnosed with TM will not develop testicular cancer at five years after the diagnosis [9], which would increase the costs of necessary exhaustive follow-ups and negatively impact health systems. Therefore, follow-up should be reserved only for the population with classic TM and risk factors for testicular malignancy [2,9].

Another significant point is its extension (classic vs. limited), and its potential risk for tumor formation is unknown. A study followed 442 patients with TM for 28 months and found that two patients developed testicular tumors, both with limited TM (both with risk factors) [10]. There is a classification of TM according to the American College of Radiology. According to this, TM is defined radiologically by the number of microliths per field (<5 microliths = limited, 5-10 microliths = grade 1, 11-20 microliths = grade 2, and >20 microliths = grade 3), and there is no significant difference in the prevalence of tumor between grades of TM [11-15].

To date, only Sharmeen et al. [12] have managed to show an association between patients with testicular cancer and the presence/absence of microliths to predict histological subtypes and have reported an association between TM and pure seminoma and a negative correlation with embryonal carcinomas; our study did not find any association between these variables. This difference is possible due to the sample size of our study, which constitutes a limitation.

Our study showed no association between the type of testicular cancer and the presence of TM, therefore discarding the possible prediction of a histological subtype with the presence of ultrasound-detected microlithiasis.

Previous studies have shown a clear association between microlithiasis and testicular cancer, particularly in patients with risk factors such as cryptorchidism, Down’s syndrome, testicular atrophy, infertility, and family history of testicular cancer [2]. However, neither follow-up nor self-examination is recommended in patients with microlithiasis without any risk factors because there are no benefits from them [15-18].

Our study is the first to report on Hispanic patients and establish the association between histological subtype and the presence of TM. Although the Hispanic population has not been associated with a specific risk factor for testicular cancer besides it ethnicity, there is evidence that prevalence and incidence is different between different ethnic groups, with non-Hispanic whites having the highest incidence and prevalence, followed by Hispanic whites, Blacks, Asians, and Asian Pacific Islanders [19,20].

## Conclusions

There was no association between TM and the histological subtype of testicular cancer in our study. As mentioned before, we recommend conducting a more extensive study to provide further scientific evidence to establish a reliable association between microlithiasis and the subtype of testicular cancer since there is a discrepancy in the results of our study with the information previously reported. We encourage the study of characterization of risk factors among ethnic groups as this field has not been explored yet.
